# A large retroperitoneal lymphatic malformation successfully treated with traditional Japanese Kampo medicine in combination with surgery

**DOI:** 10.1186/s40792-017-0358-3

**Published:** 2017-07-17

**Authors:** Toko Shinkai, Kouji Masumoto, Fumiko Chiba, Nao Tanaka

**Affiliations:** Department of Pediatric Surgery, Faculty of Medicine, University of Tsukuba, 1-1-1 Tennodai, Tsukuba, 305-8575 Ibaraki Japan

**Keywords:** Lymphatic malformations, Lymphangioma, Eppikajyutsuto, Herbal medicine, Kampo

## Abstract

**Background:**

Current treatment options for lymphatic malformations (LMs) are multimodal. Recently, the effectiveness of treating LMs with Eppikajyutsuto (TJ-28) has been reported. TJ-28 is a kind of oral herbal medicine classified as the traditional Japanese Kampo medicine.

**Case presentation:**

A 12-year-old girl was admitted to our hospital for intermittent upper abdominal pain. Radiological examinations revealed a large (9.5 × 5.8 × 10.0 cm) retroperitoneal LM, which was suspected to adhering and stretching both pancreas head and duodenum. The large retroperitoneal tumor resection might induce involving complications because of the size and the location. Therefore, we used TJ-28 in order to diminish the tumor size before surgery. The patient received oral doses of 7.5 g/day (2.5 g × 3 times/day) of TJ-28. Six months after the medication, the tumor decreased markedly to 3.5 × 1.5 × 1.2 cm in size. Thereafter, the mass was sub-totally resected (95%) via a 3 cm trans-umbilical incision without any surgical complications.

**Conclusions:**

We reported a case of successfully treated retroperitoneal LM with the combination treatment of TJ-28 and surgery. Based on our experience, this TJ-28 treatment option may be very useful in treating cases of LMs having surgical difficulties because of size and/or location.

## Background

Lymphatic malformations (LMs) usually appear as soft compressive masses at birth and are found in all age groups and in various parts of the body. LMs develop in various spectrums from localized masses to diffuse infiltration. Morphological types of LMs are categorized according to size and location, i.e., macrocystic, microcystic, mixed macrocystic and microcystic, and diffuse types [1, 2]. Although LMs are benign malformation, sometimes, its treatment can be challenging, depending on the tumor size and location. Current multimodal treatments include observation, sclerotherapy, radiofrequency ablation, laser therapy, and surgery [2]. Recently, oral medications such as sildenafil, propranolol, and sirolimus are shown to be effective for treating LMs [3–6]. In addition, in Japan, the most common treatment options include sclerotherapy using OK-432 and/or surgical resection. Recently, several investigators have reported on the effectiveness of Eppikajyutsuto (TJ-28) in treating LMs [7, 8], which is a Japanese Kampo medicine manufactured by Tsumura & Co. (Tokyo, Japan). We experienced a successfully treated case of large retroperitoneal LMs with the combination treatment of TJ-28 and surgery. Therefore, we herein reported the clinical course of this case and showed the effectiveness of our treatment.

## Case presentation

**Fig. 1:**
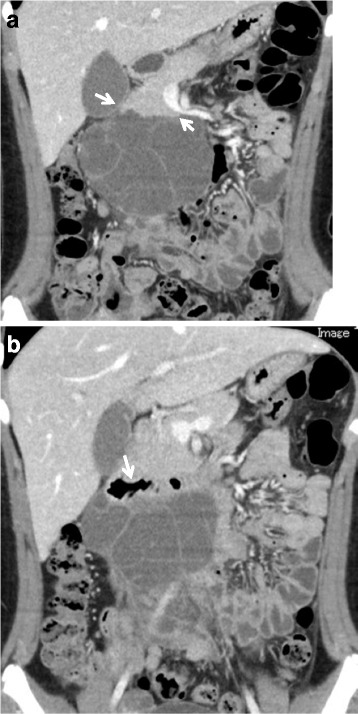
Contrast CT revealed a well-circumscribed multicystic mass measuring 9.5 × 5.8 × 10.0 cm in the retroperitoneal region. The mass, filled with serous fluid without hemorrhage or abscess, was suspected to adhering and stretching the pancreas head (**a**, *arrow*: pancreas head) by the beak sign. The tumor was suspected to adhering the duodenum (**b**, *arrow*: duodenum)

**Fig. 2:**
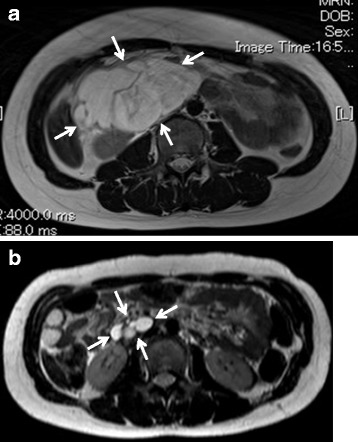
At the first admission, magnetic resonance imaging (MRI) showed that a large multicystic mass (*arrow*) revealed hyperintense on T2 images and did not contain any fat tissue (**a**). Six months after TJ-28 medication, MRI showed that the tumor size decreased markedly to 3.5 × 1.5 × 1.2 cm in size (*arrow*, **b**)

**Fig. 3:**
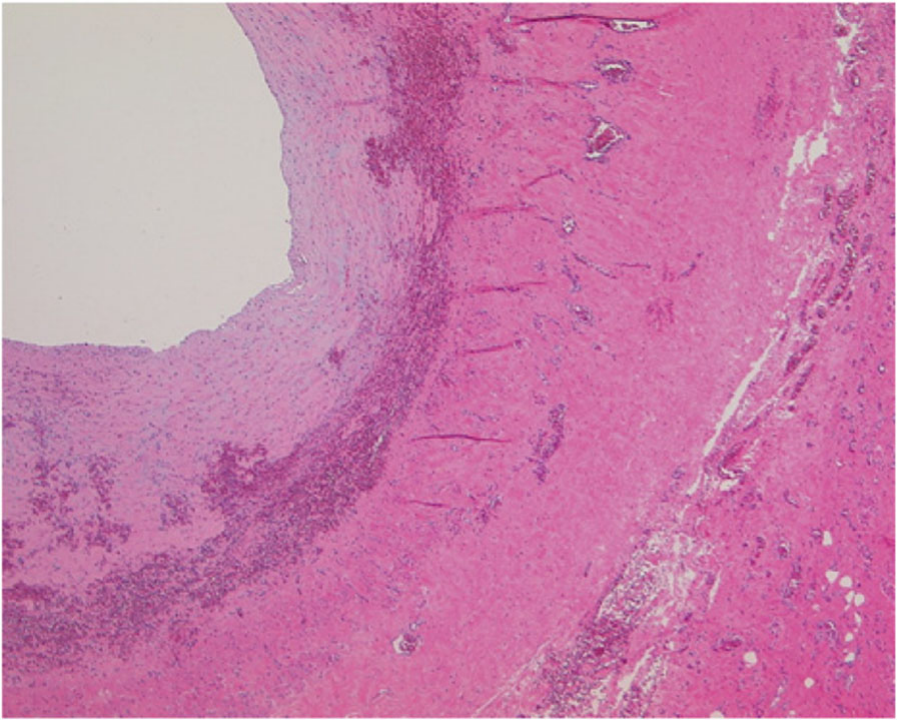
The histological findings showed that the wall of the cystic mass had hyaline degeneration and the endothelium was not existed

A 12-year-old girl was admitted to our hospital for intermittent upper abdominal pain, which continued for 3 days. The patient had no past medical histories. On admission, the patient had upper abdominal pain, nausea, and vomiting. The patient had no history associated with antecedent infections or abdominal contusions. A physical examination revealed a smooth and elastic hard mass measuring 10 cm in diameter at the center of her right flank. The patient did not have fever elevation. The patient had spontaneous pain and tenderness on and around the mass. Except for a slight elevation of CRP level, which was 1.22 mg/dl, her laboratory data indicated normal and the levels of tumor makers were also within the normal range. Ultrasound sonography (US) showed a large mass consisting of multi-macrocystic lesions. Contrast CT revealed a well-circumscribed multicystic mass measuring 9.5 × 5.8 × 10.0 cm in the retroperitoneal region. The mass, which is filled with serous fluid without hemorrhage or abscess, was suspected to adhering and stretching both pancreas head and duodenum (Fig. 1a, b). This well-circumscribed multicystic mass showed to be hypointense on T1 images and hyperintense on T2 images of magnetic resonance imaging (MRI) (Fig. 2a). The mass did not contain any fat tissue. According to the radiological findings, the mass was diagnosed as a large retroperitoneal LM. After intestinal rest therapy, her symptoms disappeared and she was discharged on the 5th day after admission. Tumor resection was considered necessary because of avoiding the recurrence of acute abdomen. However, we thought that the large retroperitoneal tumor resection might induce involving complications because of adhesion of the mass to both pancreas head and duodenum. To deal with this issue, we used a traditional Japanese Kampo medicine, Eppikajyutsuto (TJ-28), in order to diminish the tumor size before surgery. On 3 weeks after the symptoms onset, the patient received oral doses of 7.5 g/day (2.5 g × 3 times/day) of TJ-28. The patient received regular physical examination and US. On six months after the medication began, her MRI revealed that the tumor size decreased markedly to 3.5 × 1.5 × 1.2 cm in size (Fig. 2b). No side effects from TJ-28 were observed. Thereafter, we performed surgical resection, and the mass was sub-totally resected (95%) via a 3 cm trans-umbilical and longitudinal incision without any surgical complications. The cystic wall was thickened and the cystic fluid was yellowish serous with containing lymphocytes. The histological findings showed that the wall of the cystic mass had hyaline degeneration and the endothelium was not existed (Fig. 3). TJ-28 was continued for 1 month after the surgery. The patient’s postoperative course has been uneventful, and there have been no recurrences in over 3 years after the surgery.

### Discussion

Although the effectiveness of TJ-28 in treating LMs has been reported [7, 8], it is unclear that TJ-28 can completely resolve this large retroperitoneal LM. The histological findings of our case suggested that TJ-28 might have an effect of degenerating the endothelium of LMs. However, TJ-28 alone might be insufficient to complete resolution of the residual thickened sorrounding fibrous wall. Therefore, we decided to use TJ-28 combining with surgical resection.

The Japanese Kampo medicine Eppikajyutsuto (TJ-28) consists of six crude drug (herbs) components including Sekko, Mao, Sojutsu, Taiso, Kanzo, and Shokyo. TJ-28 regulates and reduces redundant body fluids and also has anti-inflammatory effects. Therefore, TJ-28 is usually used to treat edema, excessive sweating, thirst, and decreases in urine volume. In addition, recently, several investigators have reported on the effectiveness of TJ-28 in treating LMs [7, 8]. The purpose of TJ-28 usage in LMs was to regulate and reduce cystic fluids. Ogawa-Ochiai et al reported on successfully treating a case of huge mediastinal LM with TJ-28 [7]. In that case, the tumor volume was greatly reduced after TJ-28 therapy. Hashizume et al. also reported on eight cases of LMs that have been successfully treated with TJ-28 [8]. Based on their experiences, they discussed on the function of Mao (ephedra herb), which is a main component of TJ-28. Mao (ephedra herb) is suggested to work for the suppression of vascular endothelial growth factor (VEGF) activity by inhibiting prostaglandin E2 synthesis and cyclooxygenase protein synthesis [9–11]. VEGF is recognized as a key regulator of lymphangiogenesis. It has been reported that the pathogenesis of LMs is associated with various types of gene mutation of VEGF and high expression of VEGF in LMs [12]. Therefore, TJ-28 may suppress VEGF activity and reduce cystic fluids, which in turn induces LM shrinkage. In addition, this functional mechanism of TJ-28 may be considered to be similar to that of sirolimus [5, 6, 13]. According to the histological findings of the wall of the resected specimen, TJ-28 can affect on lymphatic endothelium by inducing endothelial degeneration. However, further investigation is necessary to find the pharmacological mechanism of TJ-28 to LMs.

Our objectives include realizing a better cosmetic outcome as well as minimizing surgical complications.

## Conclusions

We reported a case of successfully treated retroperitoneal LM with the combination treatment of TJ-28 and surgery. Based on our experience, this TJ-28 treatment option may be very useful in treating cases of LMs having surgical difficulties because of size and/or location.

## Abbreviations

LM: Lymphatic malformation
TJ-28: Eppikajyutsuto
VEGF: Vascular endothelial growth factor
